# Toxicity alleviation for microalgae cultivation by cationic starch addition and ammonia stripping and study on the cost assessment

**DOI:** 10.1039/c9ra03454d

**Published:** 2019-11-22

**Authors:** Jun Li, Lin Wang, Qian Lu, Wenguang Zhou

**Affiliations:** School of Resources, Environmental & Chemical Engineering, Key Laboratory of Poyang Lake Environment and Resource Utilization, Ministry of Education, Nanchang University Nanchang 330031 China luqian@ncu.edu.cn wgzhou@ncu.edu.cn

## Abstract

Aiming at promoting microalgae-based anaerobically digested swine manure (AD-SM) treatment, this work evaluated the feasibility of removing turbidity and ammonia in swine manure by cationic starch addition and air bubbling-driven ammonia stripping. It was observed that turbidity and ammonia toxicity were two main factors limiting algae growth. Addition of cationic starch effectively reduced turbidity of AD-SM by 77.10% in 40 min. 6 L min^−1^ air flow rate and 5 h stripping time were regarded as good conditions for ammonia stripping. An economic analysis was conducted to assess the feasibility of this pretreatment strategy in a pilot scale system and results indicated that unit energy input and freshwater consumption were 0.036 kW h g^−1^ dry biomass and 0.76 L g^−1^ dry biomass, respectively, much lower than those of a high dilution strategy. So it is a more promising and feasible way to pretreat AD-SM with low dilution by turbidity removal and ammonia stripping.

## Abstract 2

Aiming at promoting microalgae-based anaerobically digested swine manure (AD-SM) treatment, this work evaluated the feasibility of removing turbidity and ammonia in swine manure by cationic starch addition and air bubbling-driven ammonia stripping.

## Graphical abstract

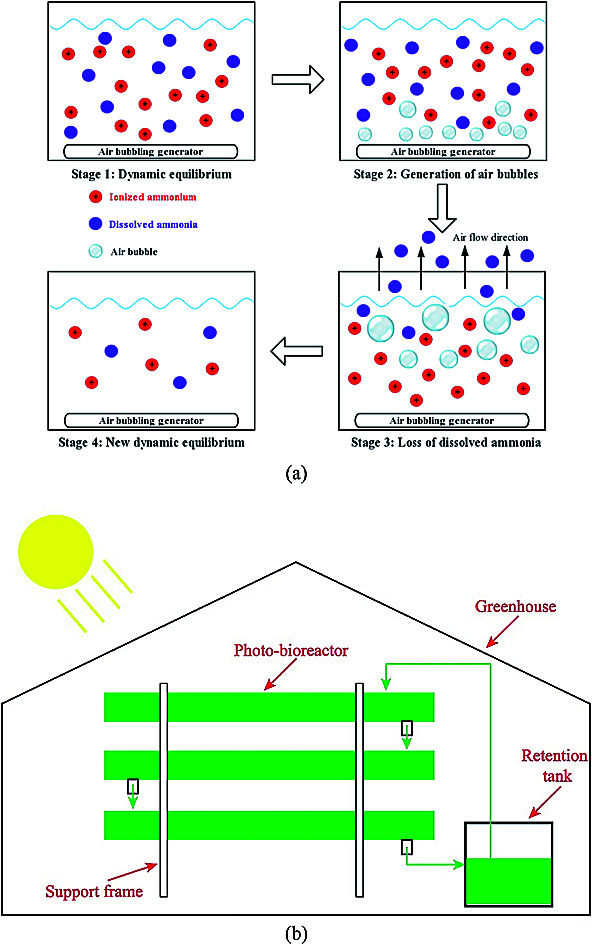

## 1 Introduction

Livestock manure, which is a type of waste generated by the animal breeding industry, has become one of the largest sources of pollution worldwide.^1^ The annual yields of manure in Europe and China even reached 1.6 and 2.5 billion tons, respectively.^2,3^ To recycle the nutrients in manure and prevent the environmental pollution, raw livestock manure could be anaerobically digested to produce biogas.^4^ It was reported that under optimized conditions, the percentage of methane in biogas produced by anaerobic digestion reached 69% and the solid content in manure was reduced by 30%.^5^ So anaerobic digestion not only generates economic benefits, but also reduces the potential pollution of manure.

Although some nutrients in manure could be removed by digestion, residual manure after anaerobic digestion may still be nutrient-rich.^6,7^ For example, the study of Demirer & Chen (2005) indicated that chemical oxygen demand (COD) in manure was only reduced by 40% after 20 days anaerobic digestion. Hence, the treatment of residual manure after anaerobic digestion is a critical issue. Through the anaerobic digestion, some solids in swine manure are converted to soluble nutrients, particularly volatile fatty acids, which are essential to microalgae growth.^8,9^ The study of Hu *et al.* (2012)^4^ demonstrated that appropriate anaerobic digestion increased the concentrations of acetic acid and propionic acid in swine manure by 50%. Since volatile fatty acids are more degradable than solid nutrients, algae grown in anaerobically digested manure have higher biomass yield than those grown in raw manure.^8^ So it is a promising idea of using microalgae to recycle the residual nutrients in anaerobically digested manure for valuable biomass production.

In the wastewater treatment plant, however, anaerobically digested manure is rarely treated by algae because of some technical challenges, which mainly include high turbidity and ammonia toxicity. Turbidity caused by the suspended solids would reduce the light transmission and further limit the photosynthesis in algal cells.^6^ Ammonia toxicity may cause intracellular oxidative stress and disturb the algal metabolisms.^10^ To use anaerobically digested manure for algae cultivation, these two challenges should be solved properly. In previous studies, anaerobically digested manure was highly diluted to reduce the contents of suspended solids and ammonia before algae inoculation.^4,8^ In the research of Wang *et al.* (2010),^6^ dilution ratio was 20-fold, meaning 19 L freshwater should be added to treat 1 L anaerobically digested manure. In some cases, the dilution ratio of AD-SM even reached 100-fold.^11^ Although high dilution pretreatment alleviates the challenges of turbidity and ammonia toxicity, it reduces the concentrations of other nutrients, such as organic carbon and phosphorus, in manure, and limits the algae growth accordingly. In addition, high consumption of freshwater would increase the treatment cost of manure. From either economic perspective or environmental perspective, it is not practically feasible to use highly diluted manure for algae cultivation.

To reduce the consumption of freshwater in the manure treatment, Deng *et al.* (2017)^12^ and Deng *et al.* (2018)^1^ conducted vacuum-assisted thermophilic anaerobic digestion and recycled some post-harvest culture broth by centrifugation. With such a pretreatment, the dilution ratio of manure for algae cultivation was reduced to 4-fold. However, thermophilic digestion, vacuum treatment, and high speed centrifugation would significantly increase the energy input and the operation cost. Because of these disadvantages, these newly developed technologies are still not feasible in the wastewater treatment plant.^12^ To pretreat the anaerobically digested manure for algae cultivation, it is essential to develop a cheap, simple, and energy-saving strategy in pilot scale system.

This study alleviated the challenges to algae growth by conducting cationic starch-assisted turbidity removal and air bubbling-driven ammonia stripping in the pretreatment of anaerobically digested swine manure (AD-SM). Cationic starch is an affordable modified starch with high flocculating capacity but no toxicity.^13^ Accordingly, algae cultivated in effluent pretreated by cationic starch could have wider usage range, including fertilizer and animal feed. In addition, compared with other ammonia removal facilities, such as vacuum thermal stripper,^14^ counter-current stripper,^15^ and steam stripper,^16^ air bubbling-driven ammonia stripper has lower cost, simpler procedure, and less energy input. Considering these advantages, the pretreatment of AD-SM by cationic starch-assisted turbidity removal and air bubbling-driven ammonia stripping should be a possible way to reduce the freshwater consumption of the algae cultivation in AD-SM.

## 2 Materials and methods

### 2.1 Swine manure and algal strain

Anaerobically digested swine manure (AD-SM), obtained from local farm in Nanchang (Jiangxi, China), was stored in refrigerator at 4 °C. In the lab scale experiment, AD-SM was sterilized at 121 °C for 30 min before algae inoculation. The 250 mL Erlenmeyer flasks with 100 mL AD-SM were used for algae cultivation. These flasks were shaken (150 rpm) under fluorescent lights (120 ± 10 μmol photons m^−2^ s^−1^) at room temperature (28 ± 1 °C).

The algal strain used for AD-SM treatment was *Chlorella vulgaris* purchased from UTEX (Texas, USA). Before inoculation into AD-SM, algae were preserved on solid artificial medium with 15% agar. Nutrient profile of artificial medium is listed as follows: NH_4_Cl (0.375 g L^−1^), K_2_HPO_4_ (0.108 g L^−1^), (HOCH_2_)_3_CNH_2_ (2.420 g L^−1^), MgSO_4_·7H_2_O (0.100 g L^−1^), KH_2_PO_4_ (0.054 g L^−1^), CaCl·2H_2_O (0.050 g L^−1^), microelements stock solution (1.0 mL L^−1^), and glacial acetic acid (1.0 mL L^−1^).^17^

### 2.2 Parameters measurement

#### 2.2.1 Nutrient profile analysis and turbidity measurement

Ammonia nitrogen (NH_3_–N), total nitrogen (TN), chemical oxygen demand (COD), and total phosphorus (TP) of AD-SM or artificial medium were measured by using analysis kits purchased from Hach Co. Ltd (USA). The measurement was performed by a spectrophotometer according to published method.^17^ Concentrations of nutrients were expressed as mg L^−1^. Nutrients removal efficiencies were calculated according to eqn (1).

1 *R* = (*N*_0_ − *N*_*t*_)/*t* × 100%

where *R* is the nutrients removal efficiencies (%); *N*_0_ and *N*_*t*_ are the concentrations of certain nutrients on day 0 and day *t*; *t* is the cultivation period (day) of algae in AD-SM.

Concentrations of short-chain fatty acids, including acetic acid, propionic acid, and butyric acid, in AD-SM were measured by using gas chromatography equipped with a flame ionization detector (GC-FID) according to the method described by Hu *et al.* (2012)^4^. Concentrations of short-chain fatty acids, expressed as mg L^−1^, were calculated based on the peak areas and the calibration curves. Turbidity meter was used to measure the turbidity, which was expressed as Nephelometric Turbidity Unit (NTU), of AD-SM. Suspended solids and pigment mainly contributed to the turbidity in wastewater.^18^

#### 2.2.2 Algae growth and biomass yield

In this work, total volatile suspended solid (TVSS), reflecting the dry weight of algae biomass, was measured according to published method.^19^ Average growth rates of algae were calculated according to eqn (2).

2 *G* = (*W*_*t*_ − *W*_0_)/*t*

where *G* is the average growth rate of algae; *W*_*t*_ and *W*_0_ are the dry weights of algae biomass on day *t* and day 0; *t* is the cultivation period (day) of algae in AD-SM.

Survival efficiency (%), which is a parameter to reflect the percentage of living algal cells in total cells, was measured with a microscope purchased from Nexcelom (USA).

#### 2.2.3 Composition of algae biomass

Harvested algae biomass was dehydrated in a vacuum dryer before protein content measurement and crude oil extraction.^20^ Protein content in algae biomass was calculated according to the total nitrogen content, which was measured by micro elemental analyzer. The nitrogen-to-protein factor (NTP) of 6.25 was used for the calculation of protein content. Detailed measurement and calculation procedures were described by Lu *et al.* (2015)^17^. To measure the oil content in algae biomass, ultrasound assisted oil extraction was performed according to previous publication and oil content of algae biomass was calculated accordingly.^19^

Chlorophyll of dry algae biomass was extracted by ethanol solvent and measured by spectrophotometer.^21^ Chlorophyll content, expressed as mg g^−1^ dry biomass, was calculated accordingly.^22^ Since chlorophyll directly participates in photosynthesis, its content could be used as a factor to reflect the growth mode of algae in AD-SM.^23^

### 2.3 Design of experiment

This work, aiming at cultivating algae in AD-SM with low dilution ratio and reducing the consumption of freshwater, consisted of four steps. First, the basic characteristics of AD-SM were measured to evaluate its feasibility for algae cultivation. Second, effects of dilution on algae growth and nutrients removal in AD-SM were assessed. Barriers to algae growth in AD-SM with low dilution ratio were identified. Third, the pretreatments, including cationic starch flocculation and ammonia stripping, were conducted to alleviate those barriers. The parameters of flocculation and stripping were optimized accordingly. Fourth, three types of manure, including raw AD-SM with low dilution, pretreated AD-SM with low dilution, and AD-SM with high dilution were compared according to the nutrients removal and biomass yield in pilot scale system. Economic analysis was conducted to assess the advantages of the integrated pretreatment developed by this work.

All the experiments and tests in this study were performed in triplicate. The results were expressed as mean ± deviation.

### 2.4 Effects of dilution on algae growth and wastewater treatment

Effects of dilution, 4, 8, 12, 16, and 20-folds, on algae growth in 5 day culture and nutrients removal in AD-SM were assessed. The optimum dilution ratio of AD-SM for algae cultivation was determined accordingly. Chlorophyll contents in algae grown in AD-SM with different dilution ratios were measured to explore the growth mode of algae in AD-SM. According to the biomass yield, chlorophyll content, and nutrients removal, some barriers to algae growth in AD-SM with different dilution ratios were identified.

### 2.5 Cationic starch flocculation and ammonia stripping

#### 2.5.1 Cationic starch flocculation

To synthesize cationic starch, 5 g corn starch was reacted with 3 g glycidyl trimethylammonium chloride (GTAC) at 60 °C in water bath for 5 hours with 1.5 mL NaOH solution (1 mol L^−1^) as catalyst.^24^ After that, excessive ethanol was added to promote the polymer sedimentation and then the precipitated polymer was dehydrated in an oven at 60 °C for 10 hours.^25,26^ Dry polymer was stored in dark at 4 °C before being used as flocculating agent for AD-SM pretreatment.

The flocculation was performed by adding certain amounts (0, 0.25, 0.50, 0.75, and 1.00 g) of cationic starch in 1 L AD-SM and mixing for 2 min. After that, AD-SM was subjected to settlement. To save energy and reduce cost, in this work, settlement was driven by gravity. Turbidities of supernatants at different settlement time were measured. After settlement, the supernatant was collected for subsequent experiment.

#### 2.5.2 Ammonia stripping

Algae were cultivated in artificial simulated medium with different ammonia concentrations (0, 100, 200, 300, 400, and 500 mg L^−1^) to evaluate the threshold of ammonia toxicity. The cultivation periods were 5 days. Expected ammonia concentration for algae cultivation was identified according to average growth rate and survival efficiency of algae.

**Fig. 1:**
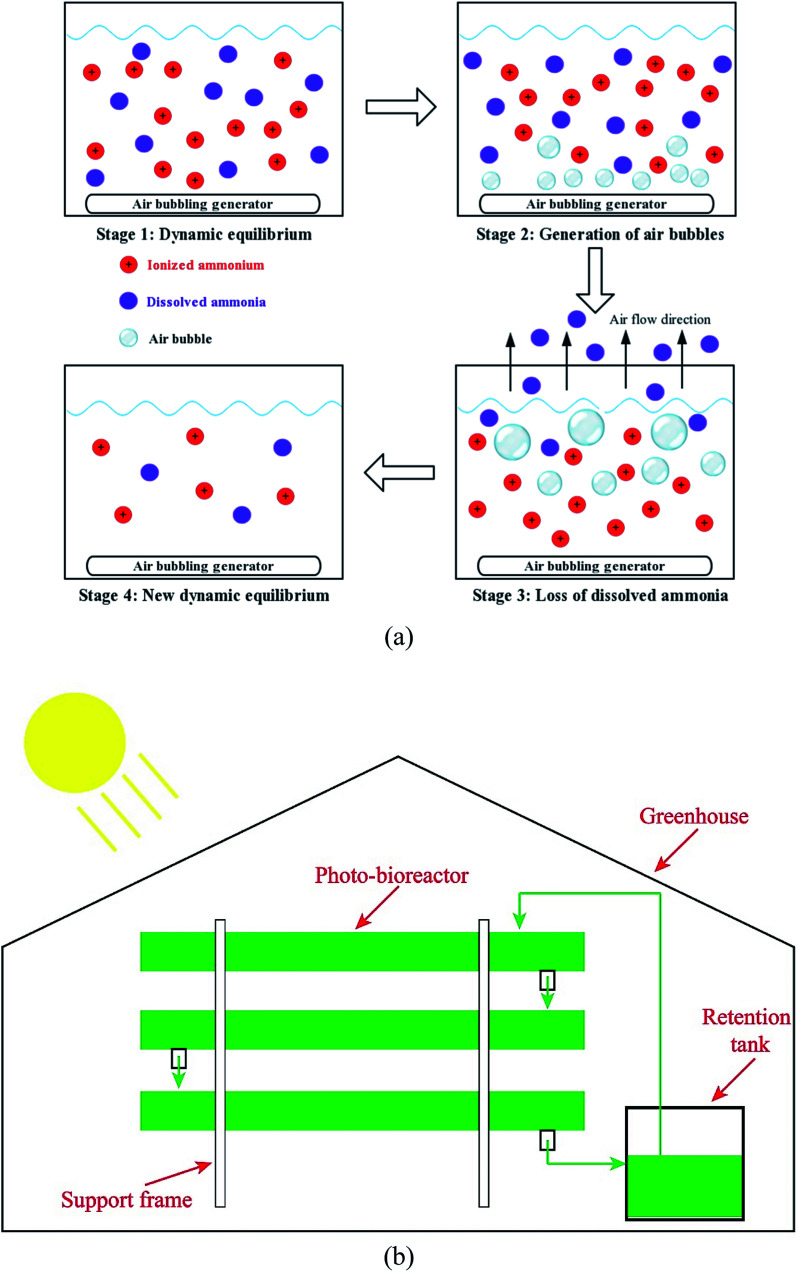
(a) Mechanisms of ammonia stripping process assisted by air bubbling; (b) diagram of the pilot-scale system for the microalgae-based manure treatment.

Air bubbling was used to strip ammonia from AD-SM with 4-fold dilution in a 2 L bottle at room temperature (28 ± 1 °C). As shown in Fig. 1(a), ionized ammonium and dissolved ammonia, two major forms of ammonia, reached dynamic equilibrium in waste effluents. Such a dynamic equilibrium is impacted by the temperature, pH value, concentrations of ions, air pressure, and some other factors.^27,28^ Under a specific condition, the ratio of ionized ammonium to dissolved ammonia is a constant. The mechanism of air bubbling-driven ammonia stripping is that air flow takes out a portion of dissolved ammonia and disturbs the dynamic equilibrium between ionized ammonium and dissolved ammonia.^29^ To reach a new dynamic equilibrium, a portion of ionized ammonium is converted to dissolved ammonia.^30^ As a result, the concentration of total ammonia/ammonium is reduced to a lower level.

The optimum stripping time was identified according to the achievement of expected ammonia concentration in AD-SM. Considering the low cost of air bubbling treatment, this method should be economically feasible in the practice.

### 2.6 Treatment of AD-SM in pilot scale system and economic analysis

Three types of AD-SM, including raw AD-SM with 4-fold dilution, pretreated AD-SM with 4-fold dilution, and AD-SM with 16-fold dilution, were used for algae cultivation in a pilot scale system (about 1500 L), consisting of a retention tank (about 300 L) and three layers of photo-bioreactors (Fig. 1(b)). This bioreactor was located in a greenhouse of which the light source was sunlight. About 700 L raw AD-SM was used to cultivate algae in each way. Considering the unfavorable conditions, such as darkness at night and temperature fluctuation, batch cultivation periods of algae in pilot scale system were extended to 8 days. Biomass yield of algae and nutrients removal in AD-SM were measured daily.

Facility investment, time, energy input, and material input were quantitatively recorded for economic analysis.^31^ The facilities mainly included greenhouse, bioreactor, flocculation tank, and ammonia stripping device. Electricity consumption, which is the major energy input, was caused by the operation of bioreactor and some other devices. Material input included freshwater and cationic starch. According to the economic analysis, the unit costs and energy inputs of algae cultivation in three types of AD-SM were calculated and compared.

## 3 Results and discussion

### 3.1 Characteristics of AD-SM

As shown in Table 1, compared with artificial medium, AD-SM from farm contained much more essential nutrients for algae growth. Concentrations of NH_3_–N, TN, TP and COD in AD-SM were 1795.04%, 568.05%, 67.42%, and 155.17% higher than those in artificial medium, respectively. In addition, in both AD-SM and artificial medium, the dominant organic carbon was acetate, which is a good carbon source for algae growth in heterotrophic/mixotrophic mode.^32^ The neutral value of pH in swine manure is another factor that is favorable to algae cultivation.

**Table 1:** Characteristics of AD-SM and artificial medium

Parameter	AD-SM	Artificial medium
TVSS (g L^−1^)	1.645 ± 0.235	0
pH	7.82 ± 0.19	7.05 ± 0.36
NH_3_–N (mg L^−1^)	1874.9 ± 6.7	98.9 ± 3.1
TN (mg L^−1^)	2534.5 ± 15.6	379.4 ± 15.5
TP (mg L^−1^)	53.7 ± 2.6	32.1 ± 2.9
COD (mg L^−1^)	9876.2 ± 72.8	3870.4 ± 98.6
Acetic acid (mg L^−1^)	1722.75 ± 68.23	1089.52 ± 59.64
Propionic acid (mg L^−1^)	919.47 ± 38.71	0
Butyric acid (mg L^−1^)	214.85 ± 12.88	0

Although some nutrients are essential to algal metabolisms, excessive concentrations may limit algae growth or even cause the failure of algae cultivation. For example, concentration of NH_3_–N in AD-SM reached 1874.95 mg L^−1^, which was much higher than the threshold of ammonia toxicity to most algal species. Lu *et al.* (2018)^43^ reported that in artificial wastewater, algae growth was prohibited when the concentration of NH_3_–N exceeded 392 mg L^−1^. Besides ammonia toxicity, high content of suspended solids, which could seriously reduce the light transmission and further limit the photosynthesis rate of algal cells in AD-SM, might be another unfavorable factor.^33^

According to the discussion above, it was hypothesized that although AD-SM contained essential nutrients and had neutral pH value, it might not be directly used for algae cultivation due to some limiting factors.

### 3.2 Algae cultivation in diluted AD-SM

**Fig. 2:**
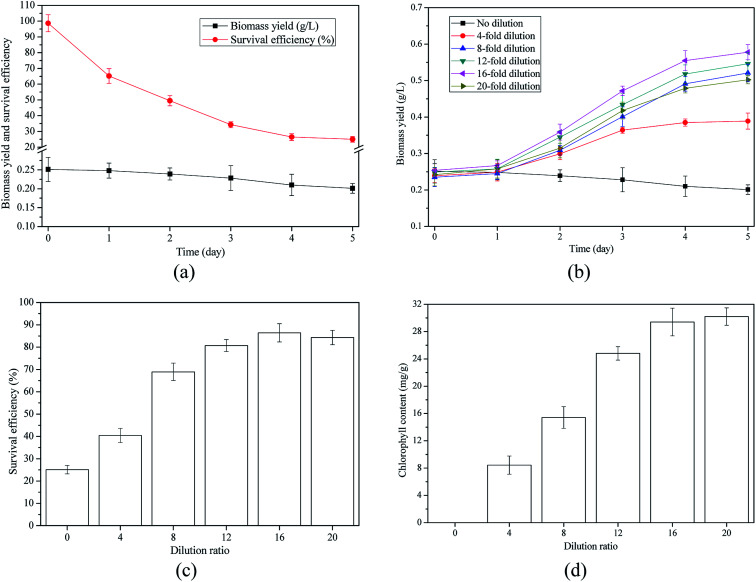
Algae growth and nutrients removal in AD-SM: (a) algae growth and survival efficiencies of cells; (b) growth of algae in AD-SM with different dilution ratios; (c) survival efficiencies of algal cells in AD-SM with different dilution ratios; (d) chlorophyll contents of algae in AD-SM with different dilution ratios.

Fig. 2(a) indicated that no algae growth was observed in raw AD-SM and the survival efficiencies of algal cells decreased gradually with the extension of cultivation period. This result confirmed the hypothesis that raw AD-SM could not be directly used for algae cultivation. To mitigate the limiting factors, in previous studies, AD-SM was diluted appropriately before algae inoculation.^6^ Hu *et al.* (2012)^4^ reported that algae had the highest biomass yield (about 0.6 g L^−1^) in AD-SM with 20-fold dilution. In some studies, the dilution ratios of AD-SM were even higher than 25-fold.^11,34^ As shown in Fig. 2(b), biomass yields of algae grown in AD-SM with 4-fold, 8-fold, 12-fold, 16-fold, and 20-fold dilution reached 0.389, 0.521, 0.546, 0.578, and 0.502 g L^−1^, respectively. In terms of biomass yield, 16-fold was the optimum dilution ratio. Not only the biomass yield, but also the survival efficiency of algal cells reached peak value (86.4%) when AD-SM was diluted by 16-fold (Fig. 2(c)). Therefore, dilution in certain range could alleviate some limiting factors in AD-SM and promote algae growth.^4,6^ Biomass yield of algae in the AD-SM with 4-fold dilution ratio was even 34.95% lower than that in the AD-SM with 16-fold dilution ratio. Hence, the specific reasons that led to the low biomass yield of algae in AD-SM should be further studied.

Fig. 2(d) indicated that chlorophyll content in algal cells increased with the dilution of AD-SM. When the dilution ratio was 4-fold, chlorophyll content was only 8.43 mg g^−1^. In AD-SM with 4-fold dilution, algal cells without sufficient chlorophyll could not have good performance in photosynthesis. Accordingly, the major carbon source for algae growth was the organic carbon from AD-SM, instead of carbon dioxide from air.^35^ High chlorophyll contents of algae in AD-SM with 16-fold and 20-fold dilution suggested that autotrophic mode was the major growth mode for algal cells in highly diluted AD-SM. The main reason for the difference in growth mode is that turbidity in AD-SM with 4-fold dilution limited the light transmission and the photosynthesis while high dilution promoted the photosynthesis.

In AD-SM with 4-fold dilution, although the nutrients were sufficient, due to the low assimilation rate of inorganic carbon by photosynthesis, biomass yield was lower than that in highly diluted AD-SM. In addition, in AD-SM with 4-fold dilution, ammonia toxicity is another limiting factor to algae growth. In some cases, excessive ammonia in wastewater or culture medium could also negatively impact the oil quality of algal biomass by causing oxidative stress.^10^ To solve the problems caused by high turbidity and ammonia toxicity, two strategies were proposed to pretreat the AD-SM. The first strategy, which has been reported by many studies, is pretreating AD-SM by high dilution.^8,11^ The second strategy is removing turbidity and ammonia in AD-SM with low dilution ratio.

### 3.3 Turbidity removal and ammonia stripping

#### 3.3.1 Turbidity removal

**Fig. 3:**
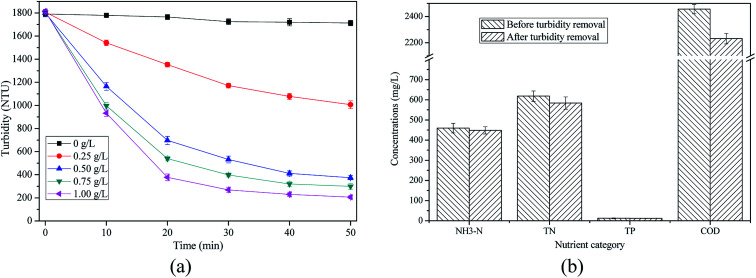
Turbidity removal by cationic starch and changes of nutrients profile: (a) changes of turbidity of supernatant; (b) concentrations of soluble nutrients in supernatant.

As shown in Fig. 3(a), turbidity of supernatant was reduced with the increase of cationic starch content. After 50 min settlement, turbidity removal efficiencies in AD-SM added with 0, 0.25, 0.50, 0.75, and 1.00 g L^−1^ cationic starch reached 4.38%, 44.35%, 79.17%, 83.42%, and 88.59%, respectively. When the cationic starch content exceeded 0.50 g L^−1^, it was not an effective and economic way to remove turbidity in AD-SM by further increasing cationic starch content. For example, residual turbidity was only reduced by 168.0 NTU when cationic starch content increased from 0.50 to 1.00 g L^−1^ (Fig. 3(a)). In addition, the residual turbidity (374.8 NTU) of AD-SM pretreated by 0.50 g L^−1^ cationic starch was low enough to support the algae growth.^36^ Therefore, the content of cationic starch for turbidity removal was set as 0.50 g L^−1^.

**Fig. 4:**
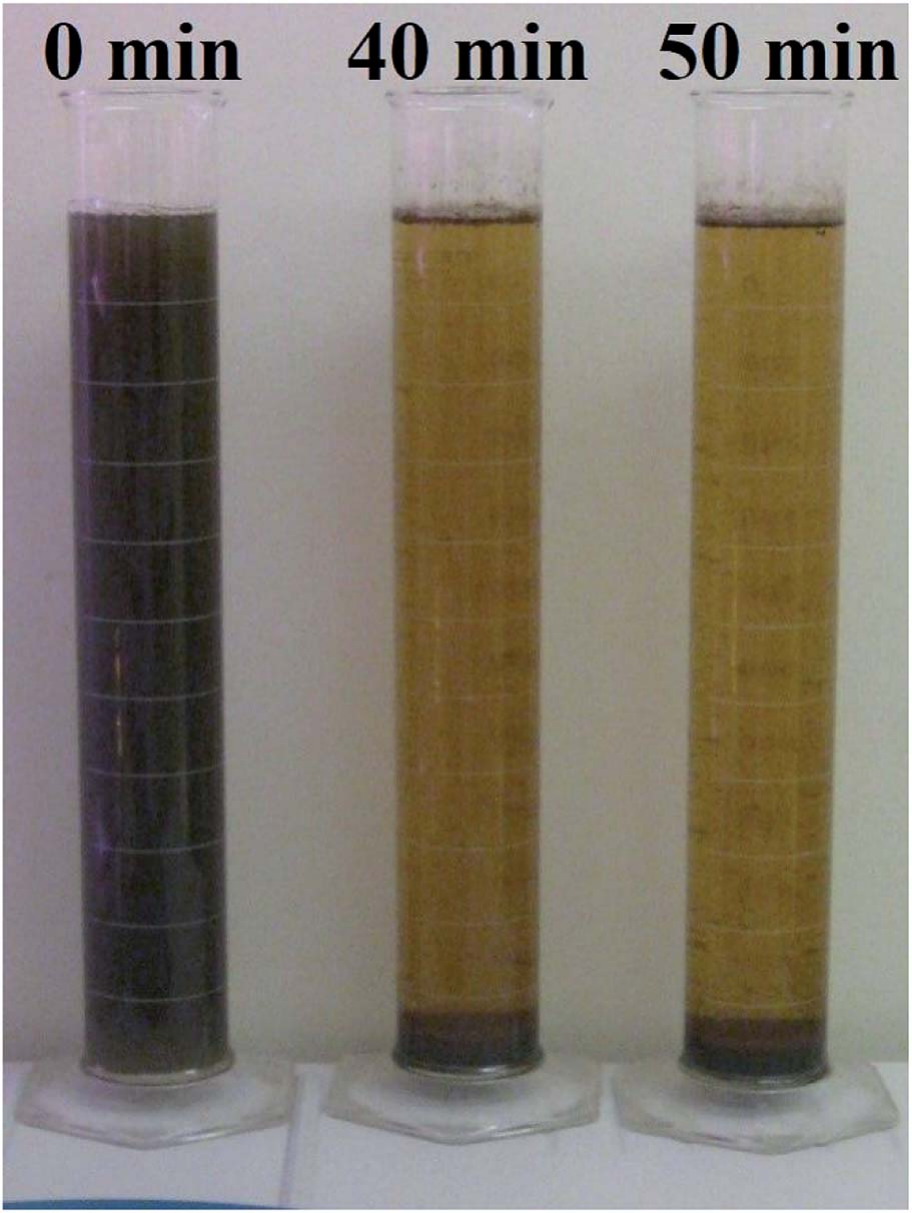
Picture of AD-SM added with 0.50 g L^−1^ cationic starch at different settlement time.

Fig. 3(a) showed that in AD-SM added with 0.5 g L^−1^ cationic starch, turbidity of supernatant decreased by 77.10% in 40 min while only decreased by 2.06% between 40 min and 50 min. This result is also supported by Fig. 4. Hence, the settlement time of turbidity removal was set as 40 min. Fig. 3(b) showed that the nutrients profile of supernatant was only changed slightly after turbidity removal, suggesting that turbidity removal by cationic starch mainly caused the settlement of suspended solids while did not remove the soluble nutrients.

Flocculating agents have been widely used to reduce turbidity in wastewater.^4,37^ In previous studies, commonly used flocculants with low expense included aluminum sulfate, poly aluminum chloride, and polyacrylamide.^38,39^ The flocculating functions were mainly expressed in two ways, combining suspended particles by functional groups or/and reducing the repulsive force between particles by neutralizing their surface electric charge.^40,41^ However, due to the toxicity of these flocculants or their degradation products, the use of algae grown in the waste effluent after flocculation would be limited. Algae biomass contaminated by toxic flocculating agents was mainly used as feedstock to produce biofuel, instead of organic fertilizer or animal feed since the toxic components will be accumulated in food-chain and finally cause food safety problems.^13^

Starch is a cheap and non-toxic flocculating agent widely applied in wastewater pretreatment. Hydroxyl functional group on starch could promote the attachment between suspended solids and further cause sedimentation.^42^ However, starch could not accelerate flocculation by changing the electric charge density on the surface of particles in aqueous phase. To overcome this weakness, in this work, cationic starch consisting of starch and cationic groups was used for flocculation. Cationic groups could reduce the repulsive force between suspended particles and promote the flocculation process, so the flocculating capacity of cationic starch is much higher than that of normal starch.^40^ In addition, algae harvested from AD-SM with cationic starch could be exploited for the production of organic fertilizer or animal feed, which have much higher profits than biofuel.^13^ Compared with the pretreatment by using freshwater or toxic flocculating agents, cationic starch-assisted turbidity removal would create more economic benefits and environmental benefits.

#### 3.3.2 Ammonia stripping

**Fig. 5:**
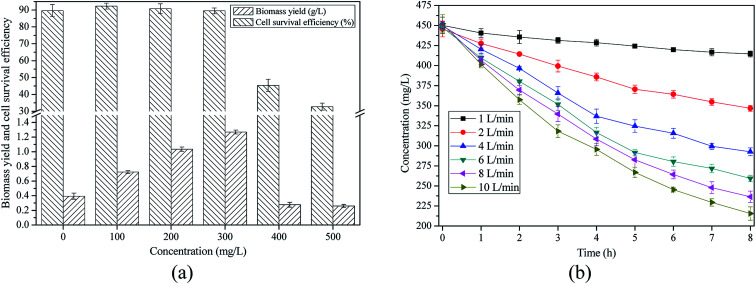
Ammonia stripping to mitigate ammonia toxicity in AD-SM: (a) identification of expected ammonia concentration in AD-SM; (b) changes of ammonia concentrations in AD-SM in stripping process.

The expected ammonia concentration was identified according to Fig. 5(a), which showed that algae had the highest growth rate (0.204 g per L per day) when the concentration of ammonia was 300 mg L^−1^. This result was in accordance with the optimum concentration of ammonia reported by previous study.^43^ In addition, survival efficiency of algal cells dropped when the concentration of ammonia exceeded 300 mg L^−1^. Therefore, in this work, the expected ammonia concentration for algae growth was 300 mg L^−1^. The purpose of ammonia stripping was to reduce the concentration of ammonia in AD-SM with 4-fold dilution to 300 mg L^−1^.

Fig. 5(b) indicated that both air flow rate and stripping time impacted the removal of ammonia in AD-SM. Although ammonia volatilization was accelerated with the increase of air flow rate, ammonia removal efficiency and air flow rate were not in a unary linear regression relationship. Removal efficiencies of ammonia reached 7.91%, 22.21%, 34.91%, 42.05%, 47.62%, and 51.32%, respectively, when the air flow rates were 1, 2, 4, 6, 8, and 10 L min^−1^. This result suggested that when the air flow rate exceeded 6 L min^−1^, the increase of ammonia removal efficiency slowed down. To reduce the energy consumption, hence, the air flow rate for ammonia stripping was set as 6 L min^−1^. It was also observed that the removal of ammonia mainly occurred in the first 4 hours (Fig. 5(b)). For example, when the air flow rate was 6 L min^−1^, 29.27% of ammonia was removed from 0–4 h while only 12.78% of ammonia was removed from 4–8 h. The main reason is that in aqueous phase with higher concentration of total ammonia in certain range, more ammonia was in the form of dissolved ammonia. Accordingly, ammonia was removed in a more efficient way during first four hours. With the decrease of ammonia concentration, the air bubbling treatment took out much less ammonia from AD-SM and the removal efficiency was reduced. Similar phenomenon was also reported in previous studies that stripped ammonia from landfill leachates and anaerobic fermentation wastewater by air bubbling.^27,44^ As shown in Fig. 5(b), to reduce the concentration of ammonia in AD-SM with 4-fold dilution to 300 mg L^−1^, stripping time should be controlled at 5 h.

According to the discussion above, pretreatment conditions for AD-SM were: 0.50 g L^−1^ cationic starch and 40 min settlement for turbidity removal and 6 L min^−1^ air flow rate for ammonia stripping (5 h).

### 3.4 Algae cultivation in pretreated AD-SM with 4-fold dilution

#### 3.4.1 Algae growth and nutrients removal

**Fig. 6:**
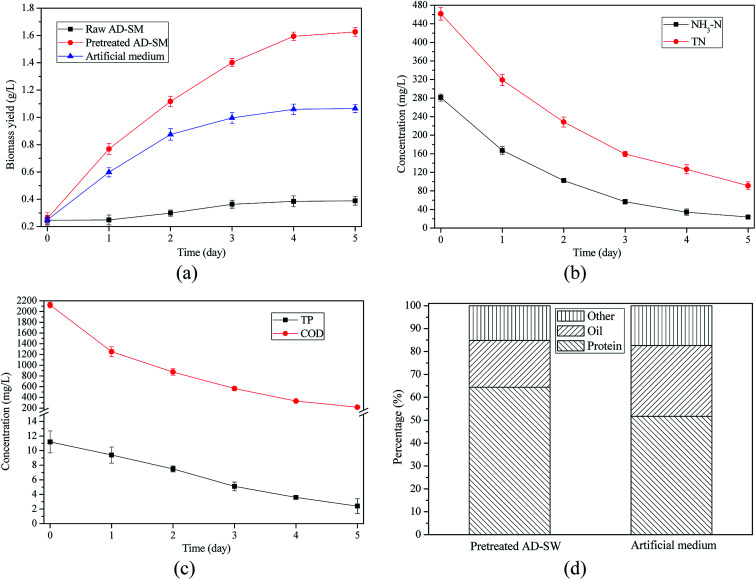
Algae growth and nutrients removal in AD-SM with 4-fold dilution and artificial medium: (a) biomass yield of algae grown in AD-SM and artificial medium; (b) removal of NH_3_–N and TN in pretreated AD-SM; (c) removal of TP and COD in pretreated AD-SM; (d) compositions of harvested algal biomass.

Fig. 6(a) showed that biomass yield in pretreated AD-SM was 266.58% higher than that in raw AD-SM. With the mitigation of barriers, biomass yield (1.626 g L^−1^) in pretreated AD-SM with 4-fold dilution was even much higher than that (1.065 g L^−1^) in artificial medium. Therefore, pretreatment by turbidity removal and ammonia stripping effectively promoted the algae growth, making AD-SM a better effluent for biomass production than artificial medium.

As shown in Fig. 6(b) and (c), removal efficiencies of NH_3_–N, TN, TP, and COD in pretreated AD-SM with 4-fold dilution reached 91.57%, 80.24%, 78.57%, and 89.74%, respectively. Removal efficiencies of NH_3_–N, TN, TP, and COD in pretreated AD-SM were 30.53%, 22.46%, 13.42%, and 21.71%, respectively, higher than those in raw AD-SM with 4-fold dilution. One of the main reasons for the higher removal efficiencies is that algae with better growth in pretreated AD-SM assimilated more nutrients. At the end of cultivation, concentrations of residual NH_3_–N, TN, TP, and COD were 23.7, 91.2, 2.4, and 217.9 mg L^−1^, meeting the requirement of wastewater discharge standard.^45^ This result demonstrated that the pretreatment of AD-SM not only generated economic benefits by producing more biomass, but also generated environmental benefits by promoting nutrients recycling. (Table 2)

**Table 2:** Nutrients removal in AD-SM with different dilution ratios

	Concentration (mg L^−1^)	0	4-fold	8-fold	12-fold	16-fold	20-fold
NH_3_–N	Initial	1873.6 ± 19.9	453.6 ± 15.6	237.1 ± 12.8	152.4 ± 18.4	120.6 ± 6.3	94.8 ± 7.5
	Final	1872.8 ± 23.6	176.7 ± 8.9	31.3 ± 0.2	0	0	0
	Removal efficiency (%)	0.04	61.04	86.80	100	100	100
TN	Initial	2535.8 ± 35.4	635.9 ± 22.5	312.8 ± 14.9	209.3 ± 8.9	155.8 ± 4.6	121.4 ± 2.6
	Final	2533.9 ± 29.8	268.5 ± 3.7	41.8 ± 4.9	12.7 ± 1.2	10.4 ± 0.9	7.6 ± 1.7
	Removal efficiency (%)	0.07	57.78	86.64	93.93	93.32	93.74
TP	Initial	54.9 ± 4.7	13.2 ± 0.9	6.8 ± 0.8	4.9 ± 0.9	3.1 ± 0.7	2.2 ± 0.3
	Final	54.1 ± 3.2	4.6 ± 0.8	0	0	0	0
	Removal efficiency (%)	1.46	65.15	100	100	100	100
COD	Initial	9932.5 ± 94.5	2484.1 ± 90.7	1256.4 ± 59.2	862.3 ± 26.5	622.9 ± 18.4	490.2 ± 11.3
	Final	9929.8 ± 75.6	794.2 ± 55.8	188.6 ± 21.7	32.9 ± 5.9	0	0
	Removal efficiency (%)	0.03	68.03	84.99	96.18	100	100

#### 3.4.2 Composition of algal biomass

Algae biomass harvested from the pretreated AD-SM with 4-fold dilution contained 64.4% protein and 20.4% oil (Fig. 6(d)). Due to the high protein content, the algae biomass could be exploited as animal feed or bio-fertilizer. Compared with the biomass harvested from artificial medium, the biomass from pretreated AD-SM contained more protein but less oil. The main reason is that ammonia concentration in pretreated AD-SM was about 187.04% higher than that in artificial medium. Sufficient ammonia was favorable to the protein synthesis in algal cells. In addition, in artificial medium with lower turbidity, algal cells had better performance in photosynthesis, which is one of the main pathways for oil synthesis.^46^ Therefore, algae biomass harvested from pretreated AD-SM and artificial medium had different nutrient compositions.

### 3.5 Comparison of pretreatment strategies and economic analysis

#### 3.5.1 Algae cultivation in pilot scale system

In pilot scale system, biomass of algae grown in raw AD-SM with 4-fold dilution, pretreated AD-SM with 4-fold dilution, and AD-SM with 16-fold dilution reached 0.347, 1.626, and 0.532, respectively (Table 3). In terms of biomass yield, pretreated AD-SM with 4-fold dilution was the best one for algae cultivation. Interestingly, it was observed that in each type of AD-SM, biomass yield in pilot scale system was lower than that in lab scale experiment. For example, in pretreated AD-SM with 4-fold dilution, biomass yield of algae grown in lab was higher than that of algae grown in pilot scale system. Similar phenomenon was also reported by previous study.^47^ Generally, some unfavorable conditions in greenhouse were the main reasons for the lower biomass yield.

**Table 3:** Algae growth and nutrients removal in pilot scale system with three types of AD-SM

Items	Raw AD-SM with 4-fold dilution		Pretreated AD-SM with 4-fold dilution		AD-SM with 16-fold dilution	
	Residual (mg L^−1^)	Removal efficiency	Residual (mg L^−1^)	Removal efficiency	Residual (mg L^−1^)	Removal efficiency
NH_3_–N	243.7	46.27%	27.8	90.12%	0	100%
TN	332.8	47.66%	141.6	69.32%	23.6	84.85%
TP	5.9	48.70%	1.2	89.29%	0	100%
COD	1134.5	54.33%	294.6	86.13%	23.5	96.23%
Biomass yield (g L^−1^)	0.347	1.597	0.532			
Result of wastewater treatment	Not dischargeable	Dischargeable	Dischargeable			

Table 3 showed that after algae cultivation, concentrations of residual nutrients in AD-SM with 16-fold dilution were much lower than those in raw and pretreated AD-SM with 4-fold dilution. The nutrients profile of raw AD-SM did not meet the requirement of discharge standard.^45^ Although nutrients removal efficiencies in pretreated AD-SM with 4-fold dilution were not the highest, it still met the requirement of discharge standard.

#### 3.5.2 Economic analysis

Economic analysis indicated that pretreated AD-SM with 4-fold dilution was the best one for algae cultivation according to the unit energy input, cost, and freshwater consumption (Table 4). Since ammonia stripping system and flocculation tank were needed for the pretreatment, the facility cost ($7549) of using pretreated AD-SM with 4-fold dilution was slightly higher than the costs of using other two types of AD-SM. In the research of Xin *et al.* (2016)^31^ that focused on the techno-economic analysis of wastewater-based biomass production, the costs of facilities, including greenhouse and bioreactor, were much higher than those reported by this work. For example, Xin *et al.* (2016)^31^ claimed that the cost of greenhouse was higher than $430000 while the cost of greenhouse in this study was only about $3400. One of the reasons for such a difference is that this study and the research of Xin *et al.* (2016)^31^ were conducted in China and United States, respectively, which have huge difference in the prices of industrial products. In addition, the different parameters of facilities used by two studies also caused the difference of cost.^48,49^

**Table 4:** Economic analysis for algae cultivation in three types of AD-SM

Items		Raw AD-SM with 4-fold dilution	Pretreated AD-SM with 4-fold dilution	AD-SM with 16-fold dilution
Facility	Greenhouse	$3476	$3476	$3476
	Bioreactor with circulation device (1500 L)	$2975	$2975	$2975
	Ammonia stripping system	—	$650	—
	Flocculation tank (500 L)	—	$448	—
	Summary	$6451	$7549	$6451
Time	Volume	2800 L	2800 L	11 200 L
	Treatment batch	2	2	8
	Time	16 days	17 days (1 day for pretreatment)	64 days
Energy input	Operation of greenhouse	25.6 kW h	25.6 kW h	102.4 kW h
	Operation of bioreactor	51.2 kW h	51.2 kW h	204.8 kW h
	Air bubbling device	—	22.0 kW h	—
	Mixing device in flocculation tank	—	0.8 kW h	—
	Summary	76.8 kW h	99.6 kW h	307.2 kW h
Material input	Cationic starch	—	1.05 kg	—
	Freshwater	2100 L	2100 L	10 500 L
Other fees	Labor salary	$350	$370	$1400
	Post-treatment	$400	—	—
	Land utilization fee	$105	$112	$420
Unit cost/input	Unit energy input	0.105 kW h g^−1^ dry biomass	0.036 kW h g^−1^ dry biomass	0.052 kW h g^−1^ dry biomass
	Unit energy cost	$0.016 g^−1^ dry biomass	$0.005 g^−1^ dry biomass	$0.008 g^−1^ dry biomass
	Unit freshwater consumption	2.88 L g^−1^ dry biomass	0.76 L g^−1^ dry biomass	1.76 L g^−1^ dry biomass

Since 700 L raw AD-SM yielded 11 200 L AD-SM at 16-fold dilution, it was necessary to treat the AD-SM in 8 batches. However, it only took 2 batches to treat the AD-SM with 4-fold dilution. Accordingly, the time (64 days) of treating AD-SM with 16-fold dilution was much longer than that (16 or 17 days) of treating AD-SM with 4-fold dilution (Table 4). In the practice, long treatment period would increase the operation cost and seriously reduce the treatment capacity of the wastewater treatment plant. Therefore, saving time is one of the great advantages of low dilution strategy for microalgae-based AD-SM treatment.

The energy consumption was mainly caused by the operation of greenhouse and bioreactor. In this work, the average electricity consumption each day was about 4.8 kW h. Total electricity consumption of the pretreatment by turbidity removal and ammonia stripping was only 22.8 kW h, so the pretreatment slightly increased the electricity consumption. Due to the long cultivation time, electricity input of AD-SM with 16-fold dilution was 208.43% higher than that of pretreated AD-SM with 4-fold dilution. Besides the energy input, low dilution effectively reduced the freshwater consumption. As shown in Table 4, the freshwater consumption of AD-SM with 4-fold dilution was only 20% of the freshwater consumption of AD-SM with 16-fold dilution. Accordingly, the cost of freshwater consumption was reduced by the low dilution strategy. Since freshwater is a valuable resource in the nature, low freshwater consumption will also reduce the footprint of algae cultivation and generate environmental benefits.^50^

Based on the data in Tables 3 and 4, it was summarized that algae cultivated in pretreated AD-SM with 4-fold dilution had the lowest unit energy cost ($0.004 per g dry biomass) and the lowest unit freshwater consumption (0.76 L g^−1^ dry biomass). Although the total energy input of using raw AD-SM was lower than that of using pretreated AD-SM, low biomass yield in raw AD-SM increased the unit energy input and unit energy cost. The unit energy input of using pretreated AD-SM with 4-fold dilution was 30.77% lower compared with that of using AD-SM with 16-fold dilution. In addition, the unit freshwater consumption of using pretreated AD-SM with 4-fold dilution was 56.81% lower than that of using AD-SM with 16-fold dilution. Therefore, it has great advantages to use pretreated AD-SM with low dilution for algae cultivation.

## 4 Conclusions

It is concluded that (1) high turbidity and ammonia toxicity are two barriers to algae growth in AD-SM with low dilution; (2) high dilution is an effective way to mitigate these barriers, but it could not be widely applied due to the high energy input, long treatment time, and high freshwater consumption; (3) combined cationic starch addition and ammonia stripping effectively removed turbidity and ammonia in AD-SM. As a result, the pretreated AD-SM was dischargeable after algae cultivation; and (4) according to the economic analysis, it has great advantages to use pretreated AD-SM with low dilution for algae cultivation.

## Conflicts of interest

There is no conflict to declare.

## Supplementary Material
